# Antimicrobial Activity of Essential-Oil-Based Nanostructured Lipid Carriers against *Campylobacter* Spp. Isolated from Chicken Carcasses

**DOI:** 10.3390/pharmaceutics16070922

**Published:** 2024-07-11

**Authors:** Henrique Machado Pires, Luciana Machado Bastos, Elenice Francisco da Silva, Belchiolina Beatriz Fonseca, Simone Sommerfeld, Robson José de Oliveira Junior, Lígia Nunes de Morais Ribeiro

**Affiliations:** 1 Institute of Biotechnology, Federal University of Uberlandia, Uberlandia 38400-902, Brazil; henriiquemp@hotmail.com (H.M.P.); luciana.bastos@ufu.br (L.M.B.); elenicefranciscodasilva@gmail.com (E.F.d.S.); robson_junr@yahoo.com.br (R.J.d.O.J.); 2 School of Veterinary Medicine, Federal University of Uberlandia, Uberlandia 38400-982, Brazil; biafonseca@ufu.br (B.B.F.); simone.sommerfeld@ufu.br (S.S.)

**Keywords:** essential oil, vegetable butter, nanocolloids, nanotoxicity, efficacy, stability

## Abstract

*Campylobacter* is a virulent Gram-negative bacterial genus mainly found in the intestines of poultry. The indiscriminate use of traditional antibiotics has led to drug resistance in these pathogens, necessitating the development of more efficient and less toxic therapies. Despite their complex biologically active structures, the clinical applications of essential oils (EOs) remain limited. Therefore, this study aimed to increase the bioavailability, stability, and biocompatibility and decrease the photodegradation and toxicity of EO using nanotechnology. The diffusion disk test revealed the potent anti-Campylobacter activity of cinnamon, lemongrass, clove, geranium, and oregano EOs (>50 mm). These were subsequently used to prepare nanostructured lipid carriers (NLCs). Formulations containing these EOs inhibited *Campylobacter* spp. growth at low concentrations (0.2 mg/mL). The particle size, polydispersity index, and zeta potential of these systems were monitored, confirming its physicochemical stability for 210 days at 25 °C. FTIR-ATR and DSC analyses confirmed excellent miscibility among the excipients, and FE-SEM elucidated a spherical shape with well-delimited contours of nanoparticles. The best NLCs were tested regarding nanotoxicity in a chicken embryo model. These results indicate that the NLC-based geranium EO is the most promising and safe system for the control and treatment of multidrug-resistant strains of *Campylobacter* spp.

## 1. Introduction

*Campylobacter* is a virulent Gram-negative bacterial genus mainly found in the intestines of poultry [1], dogs, and cats [2]. These pathogens can cause bloody diarrhea, abdominal cramps, nausea, and vomiting. In more complex cases, they can cause Guillain–Barré syndrome and death [3]. Since its first recognition, several pathogenic species of *Campylobacter* that cause human campylobacteriosis have been cataloged using phylogenetic tools [4]. *Campylobacter jejuni* is the main cause of foodborne infections in the United States, with 1.5 million people affected each year [5]. The indiscriminate use of traditional antibiotics, such as ciprofloxacin and other fluoroquinolones, has led to drug resistance in these virulent pathogens. Thus, *Campylobacter*-related infections remain a public health issue [6]. The *Campylobacter* drug resistance mechanism is usually caused by mutations in their DNA gyrase region. A single mutation in this enzyme reduces the susceptibility of *Campylobacter* spp. strains to several drugs [4]. Therefore, the discovery of new antimicrobial compounds against *Campylobacter* ssp. strains is urgently needed to mitigate campylobacteriosis. Natural lipids from vegetables or animals, such as waxes, oils, and butters, have been used as popular medicines for thousands of years and are processed as infusions, syrups, poultices, and ointments [7]. Essential oils (EOs) are lipids derived from the secondary metabolism of vegetables, showing a complex structure with biological activity. They have fungicidal, antibiotic, antiviral, biopesticidal, and antioxidant properties [8,9,10,11,12]. EOs are mainly composed of terpenes and phenols with recognized antibiotic activity [13]. Thus, they are commonly used as candidates for treatment against several species, such as *Salmonella* ssp. [14] and *Campylobacter jejuni* [8]. However, the hydrophobicity, photosensitivity, high volatility, strongly basic pH, marked organoleptic properties, and hydrolytic ability of EOs limit their applications [15]. Nanoencapsulation is an alternative tool for preventing photodegradation, altering physicochemical properties, and increasing bioavailability, thereby optimizing the efficacy of EOs [16]. Nanotechnology is an innovative tool for developing nanostructured formulations for different applications [17]. 

Different nanocarriers encapsulating natural lipids for several applications have been reported. Liposomes encapsulating EO to assess their fungicidal [18] and bactericidal [19] activities have been described. Additionally, solid lipid nanoparticles (SLNs) have been investigated as antioxidants [20], and lipid nanoparticles loaded with essential oils have showed antimicrobial activity against pathogenic bacteria [21] and anticancer properties [22].

Nanostructured lipid carriers (NLCs) are composed of a blend of two or more solid and liquid lipids stabilized by surfactants at room temperature. These systems are highly efficient in the encapsulation of water-insoluble molecules, prolong the release of active compounds, and have excellent physicochemical stability [23]. Thus, they can be produced on a large scale and are promising alternatives for the delivery of antimicrobials [24]. Natural lipid matrices formed by butters, waxes, and EOs with strong antimicrobial activity within NLC can play structural and bioactive roles in the system. They can act as components of nanoparticles and as antimicrobial agents [8]. This dual role increases bioavailability, decreases toxicity, and prevents the degradation of EO, which are the main factors that limit their use. This work described the development of novel NLC formulations composed of natural lipids with antimicrobial activity against strains of *Campylobacter* ssp. The main goals of this work were to increase the bioavailability, stability, and biocompatibility and decrease the photodegradation and toxicity of EO. Our findings could advance a clinical application of these lipids in the near future.

## 2. Materials and Methods

### 2.1. Bacterial Strains Inoculation

Three *Campylobacter jejuni* strains (64/5, 30/1, and 34,763/3) and three *Campylobacter coli* strains (131/5, 131/6, and 131/7) were extracted from chicken carcasses. For all microbial susceptibility tests, bacterial strains were inoculated onto blood agar plates fortified with Ca^2+^ and Mg^2+^ and 5% sheep blood (Laborclin^®^, Pinhais, Brazil) and incubated at 42 ± 1 °C in microaerophilic conditions for 48 h. After 2 days, isolated colonies of 3–5 species of the same morphological type were collected and dispersed in 1 mL of sterile saline solution (0.9%) until reaching a final concentration of 1.5 × 10^8^ colony-forming units (CFUs) mL^−1^, with the inoculum corresponding to 0.5 MacFarland scale turbidity.

### 2.2. Screening of EO

Lemongrass, cinnamon, geranium, clove, oregano, tea tree, sandalwood, citronella, thyme, copaiba, garlic, and lavender EO, as well as two vegetables, avocado, and aloe vera oils (Engenharia das Essências^®^, São Paulo, Brazil) were subjected to in vitro antimicrobial susceptibility testing using diffusion disk test. Then, plates containing blood agar were fortified with Ca^2+^ and Mg^2+^ and 5% sheep blood (Laborclin^®^) under sterile conditions. The inoculum of *Campylobacter* strains was evenly distributed on the agar surface using a sterile swab and allowed to stand at room temperature for approximately 5 min. Next, disks were placed under the agar and 35 µL of each EO were added. The plates were incubated in an oven at 42 ± 1 °C for 48 h under microaerophilic conditions [25]. The growth inhibition zones (mm) were then measured in duplicate.

### 2.3. Preparation of NLC Formulations

Different formulations were prepared using the hot emulsification–ultrasonication method. The lipid phases of the formulations consisted of various natural lipids (Table 1). All lipid phases were heated in a water bath 10 °C above the melting temperature of each solid lipid (Cocoa butter—45 °C; beeswax—64 °C; Murumuru butter—47 °C). The aqueous phase, which was the same for all formulations, was composed of a poloxamer solution (5% *w*/*v*, Sigma Aldrich^®^, St. Louis, MO, USA) that was heated to the lipid phase at the same temperature. For pre-emulsion formation, the aqueous phase was added dropwise to each lipid phase while stirring at 10,000 rpm for 2 min in an Ultra-Turrax homogenizer (Ultra-Turrax^®^ T18, Berlin, Germany). The resulting pre-emulsions were immediately sonicated for 15 min. Next, the formed nanoemulsions were cooled in an ice bath until reaching 25 °C to solidify the formed nanoparticles [18].

### 2.4. Physicochemical Stability Study

Particle size (nm), polydispersity index (PDI), and Zeta potential (mV) measurements of NLC and NLC-based EO formulations were determined by the dynamic light scattering technique. The formulations were diluted (1:1000 *v*/*v*) in deionized water and analyzed using LiteSizer 500 equipment (Anton Paar, Berlin, Germany). The same parameters were followed in triplicate for 210 days (25 °C) [26]. One-way analysis of variance (ANOVA) and Tukey’s post hoc statistical tests were used to determine intragroup statistical differences over time. *p* < 0.05 was considered statistically significant.

### 2.5. Determination of the Minimum Inhibitory Concentration (MIC) of Nanostructured Lipid Carriers

The NLC formulations were evaluated by the determination of the MIC of different *Campylobacter* strains. The experiment was performed in 96-well plates in triplicate, and the bacterial suspension was diluted to a final concentration of 1 × 10^5^ CFU⋅mL^−1^ per well. Different concentrations of the formulations were then added to the 96-well plates to a final volume of 0.1 mL. Mueller Hilton Broth (Biolog^®^, São Paulo, Brazil) was fortified with Ca^2+^ and Mg^2+^ and 5% sheep blood (Laborclin^®^), and the inoculum was added to a final volume of 0.1 mL comprising 1 × 10^5^ CFU⋅mL^−1^ of bacteria to prepare the position control. A negative control was prepared without bacteria. The 96-well plates were incubated at 42 °C for 48 h under microaerophilic conditions [24]. MIC values were determined for each NLC formulation. A t-test was used to evaluate intergroup statistical differences. *p* < 0.05 was considered statistically significant.

### 2.6. Structural Characterization

The structural characterization of nanoformulations and their excipients was performed by Field-Emission Scanning Electron Microscopy (FE-SEM), infrared spectroscopy (FTIR-ATR), and differential scanning calorimetry (DSC) analyses.

The nanoparticle morphologies of NLC and NLC-based EO samples were elucidated using FE-SEM. A drop of each sample was added to a glass coverslip nailed to an aluminum stub. After the complete evaporation of the solvent, the stubs were subjected to sputtering for 120 s at 30 kV and stored in a dissector until further analysis. The samples were observed using a Tescan VEJA 3 LMU FE-SEM with secondary and backscattered electron detectors operating in high vacuum under a voltage of 20 kV.

The spectral range was 650 to 4000 cm^−1^, with resolution of 2 cm^−1^ in the FTIR-ATR analyses. The DSC analyzes were carried out in a nitrogen atmosphere, at a flow rate of 50 mL/min^−1^, in the temperature range from 0 to 100 °C, at a heating rate of 10 °C/min. All samples were added in sealed aluminum pans. TA equipment, model Q20, was used for these analyses.

### 2.7. In Vivo Nanotoxicity Assay on Chicken Embryo Model

The nanotoxicity of the NLC formulations and their respective emulsified EO (as controls) was evaluated by the in vivo chicken embryo model according to the viability (%) and embryo (g) weight changes [23]. In total, 68 eggs of Gallus gallus (lineage W-36) were subjected to ovoscopy before the analyses to ensure that the embryos within seven days of development were alive. The eggs were weighed and divided into nine groups (*n* = 7): negative control (NC), composed of 0.85% saline solution; NLC control, no EO addition; EO emulsion, 3% *w*/*v*; and NLC, with 3% *w*/*v* EO. Next, all eggs were incubated for 72 h. Embryo mortality was analyzed daily to determine viability (%). The eggs were then weighed after 14 days of embryonic development, and the embryos were weighed after death. Changes in embryonic weight were calculated as the difference between the weight of the eggs before and after treatments, according to the following equation:

aW = (ce.ysW × 50)/ieW(1)

where aW is the egg weight adjusted to 50 g, ce.ysW is embryonic weight, and ieW is the initial egg weight. 

ANOVA and Tukey’s statistical tests were used to assess intergroup statistical differences in embryonic weight changes. *p* < 0.05 was considered statistically significant. The chi-square test was used to evaluate embryonic viability, followed by a test of the difference between the two proportions, considering the NC and all other groups. GraphPad Prism version 8 (GraphPad Software, Boston, MA, USA) was used for all statistical analyses.

## 3. Results

### 3.1. Screening of Essentials Oils

The EOs with the best antimicrobial activity against *Campylobacter* ssp. strains were used as active and structural excipients in the preparation of NLC. Of twelve EOs tested, five showed the most promising results against different strains of *Campylobacter* ssp. These were cinnamon, lemongrass, clove, geranium, and oregano EOs, which had average inhibition halos of 95.00, 93.50, 47.50, 51.00, and 92.00 mm, respectively (Table 2). Thus, they were used as liquid lipids in the composition of NLC, in addition to different solid lipids (murumuru butter, cocoa butter, and beeswax) and surfactants.

### 3.2. The In Vitro Antimicrobial Activity of Nanostructured Lipid Carriers

The EOs selected in the screening step were encapsulated in NLC, resulting in F1 (lemongrass EO and murumuru butter), F2 (cinnamon EO and cocoa butter), F3 (geranium EO and beeswax), F4 (clove EO and cocoa butter), and F5 (oregano EO and beeswax) formulations. The MIC was determined for each sample against the three strains of *C. coli* and three strains of *C. jejuni* (Table 3). All formulations showed intragroup statistically significant differences, as determined using one-way ANOVA and Tukey’s post hoc tests (*p* < 0.05). The formulations containing lemongrass (F1), cinnamon (F2), geranium (F3), clove (F4), and oregano (F5) EOs inhibited the growth of most *Campylobacter* strains at low concentrations (0.2–4.0 mg/mL^−1^). Some NLCs containing EOs, such as lemongrass and geranium EOs, inhibited *C. jejuni* strain 64/5 at highest concentrations of approximately 24.51 mg/mL^−1^ and 39.47 mg/mL^−1^, respectively. Similarly, the control formulations (F7—cocoa butter and F8—beeswax) showed antimicrobial effects against *C. coli* strains 131/5 and 131/6 at average highest concentrations of 39.21 mg/mL^−1^ and 13.16 mg/mL^−1^, respectively. Contrastingly, the formulation that only contained murumuru butter (F6) inhibited the growth of strains 131/5 and 131/6 at concentrations of 3.33 mg/mL^−1^ and 0.32 mg/mL^−1^, respectively. In short, NLCs comprising lemongrass, cinnamon, and geranium EO inhibited most multidrug-resistant *Campylobacter* strains at the lowest concentrations (approximately 0.2 mg/mL^−1^).

### 3.3. Physicochemical Stability Study

**Figure 1:**
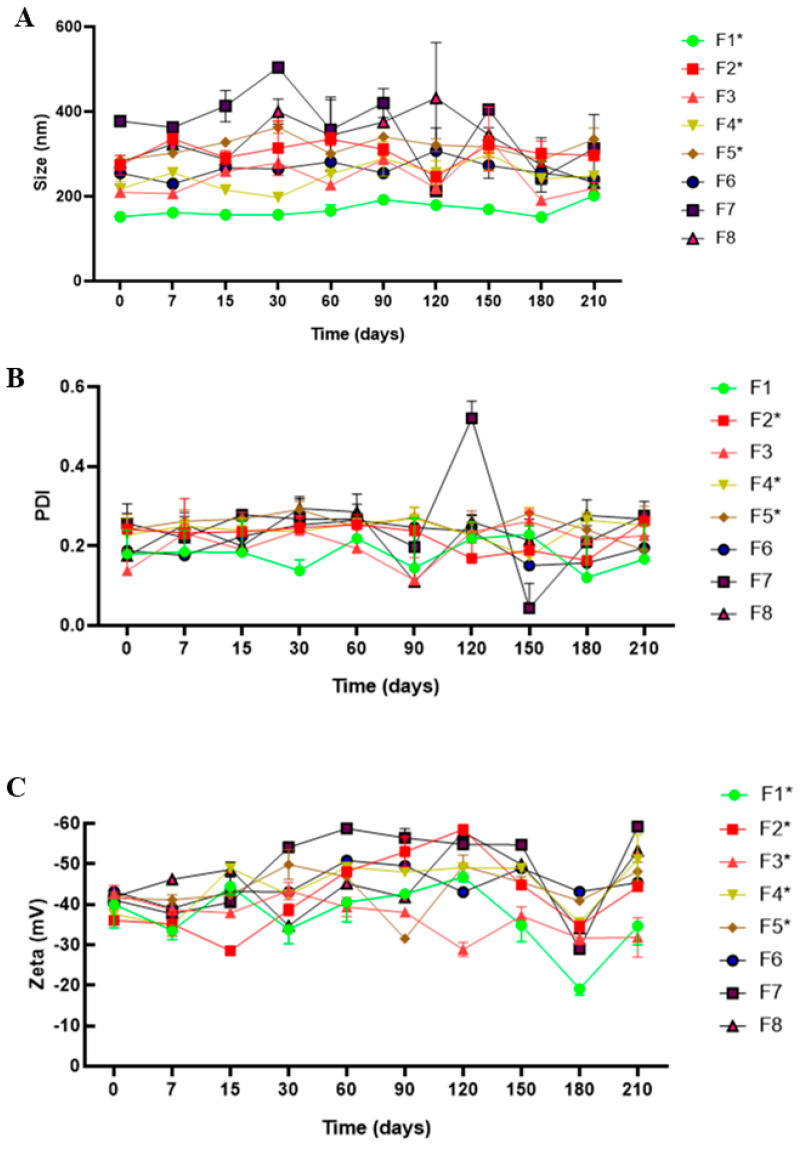
Long-term physicochemical stability of NLC formulations in terms of size (**A**), PDI (**B**), and zeta potential (**C**) values, as monitored using DLS for 210 days (25 °C); *n* = 3. One-way ANOVA and Tukey’s post hoc tests were used to analyze intragroup statistically significant differences over time; * *p* < 0.05.

Figure 1 shows the physicochemical stability of all NLC formulations. The particle size of the NLC-based EO was approximately 148.18–284.21 nm. F1, F2, and F3 showed no statistically significant differences over time, exhibiting initial and final particle sizes of approximately 151.81–179.16 nm, 245.4–231.8 nm, and 208.4–219.73 nm, respectively. Other formulations showed particle size fluctuations without evidence of instability during the analysis, as expected for nanocolloids [20]. However, the control formulations containing only the solid lipid had the highest initial and final sizes during monitoring, with F6 (NLC-based murumuru butter), F7 (NLC-based cocoa butter), and F8 (NLC-based beeswax) reaching sizes of 307.5, 512.3, and 431.8 nm, respectively, at the end of the experiment (*p* < 0.05). Most formulations had constant PDI values, with minor variations. F1 showed initial and final values of approximately 0.18 and 0.22, respectively; F3 showed 0.13 and 0.23, respectively. Similarly, F6 and F8 control formulations exhibited initial and final values of approximately 0.19–0.24 and 0.17–0.26, respectively. F7 had a final PDI value of 0.521 (*p* < 0.05). The Zeta potential values varied in this study. F1 showed initial values of −40.03 mV and −46.80 mV after 210 days; F3 exhibited initial and final values of −42.5 and −28.83 mV, respectively. The NLC controls F6 and F8 had initial and final values of −43.13 and −43.00 mV and −41.8 and −33.41 mV, respectively. In contrast, F7 showed the highest zeta potential values, with initial and final values of −41.07 and −54.83 mV, respectively.

### 3.4. Structural Characterization 

**Figure 2:**
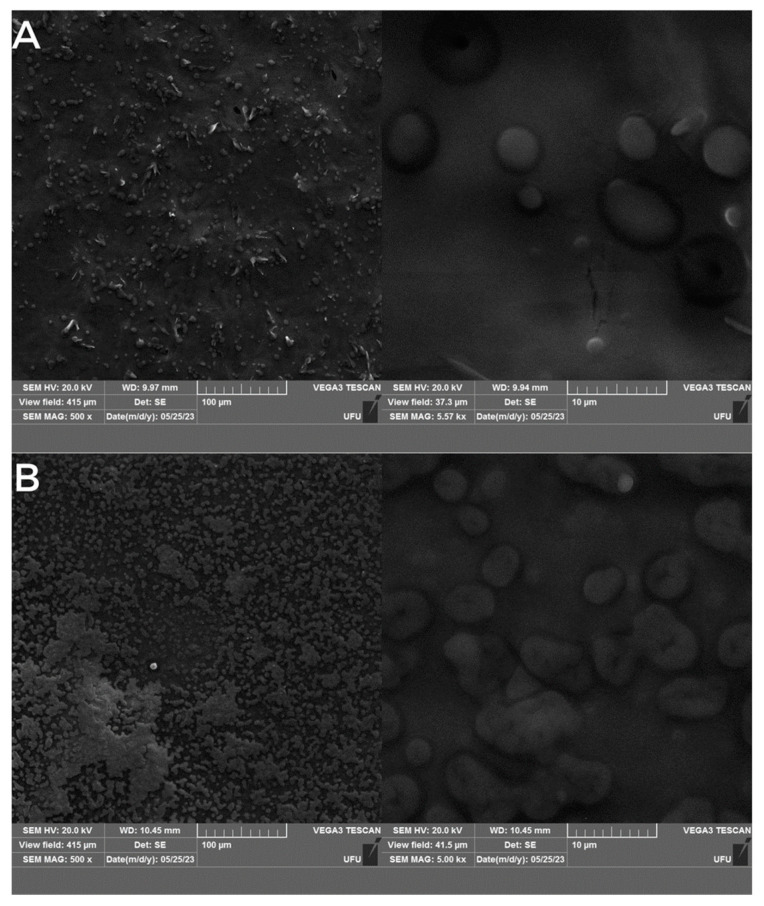
FE-SEM images of NLC (**A**) and respective NLC control (**B**) at 500× (**left**) and 5000× (**right**) magnifications.

The morphological features of all formulations were elucidated using FE-SEM (Figure 2). All NLC formulations exhibited spherical shapes with visible contours, as expected for this system [17].

**Figure 3:**
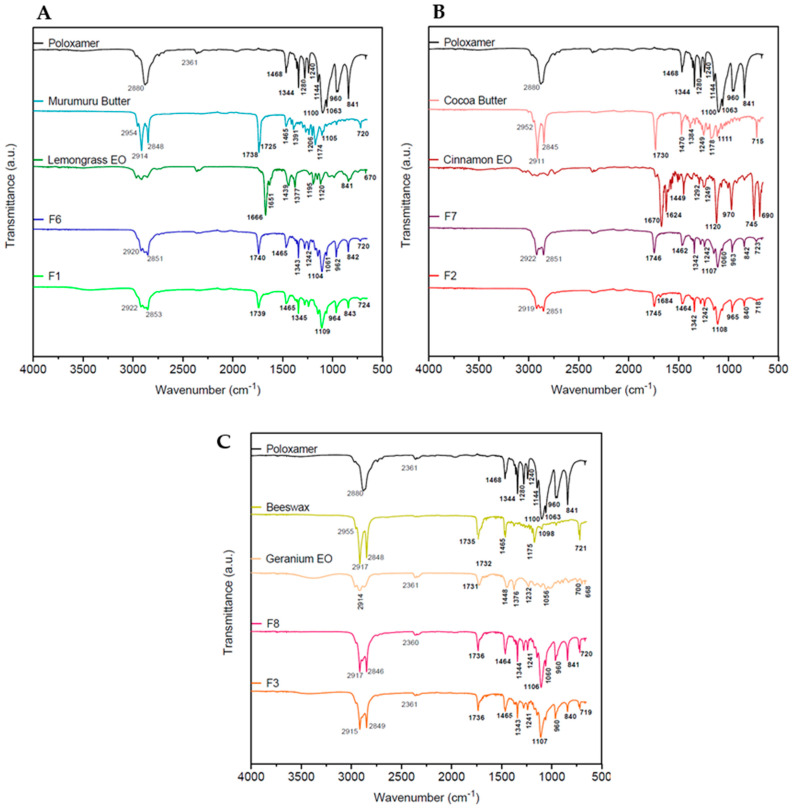
FTIR−ATR spectra of F1 (**A**), F2 (**B**), F3 (**C**), and their respective controls and excipients.

The FTIR-ATR technique allows understanding of the interactions between the excipients and bioactive compounds used in formulations. As expected, the spectra of F1 (Figure 3A), F2 (Figure 3B), and F3 (Figure 3C) formulations showed overlapped bands of poloxamer and their solid lipids: murumuru butter, cocoa butter, and beeswax, respectively. Typical bands of its lipid components were revealed in the regions between 2846 and 2922 cm^−1^ (O-CH_2_ and CH) and 1735–1746 cm^−1^ (C = O) [22]. The surfactant showed typical bands of aliphatic chain ethers at approximately 2880 and 1344 cm^−1^, associated with O-CH_2_ and O-C-O vibrations, respectively [18]. F1, F2, and F3 have exhibited stretching vibrations associated with their respective F6, F7, and F8 controls.

**Figure 4:**
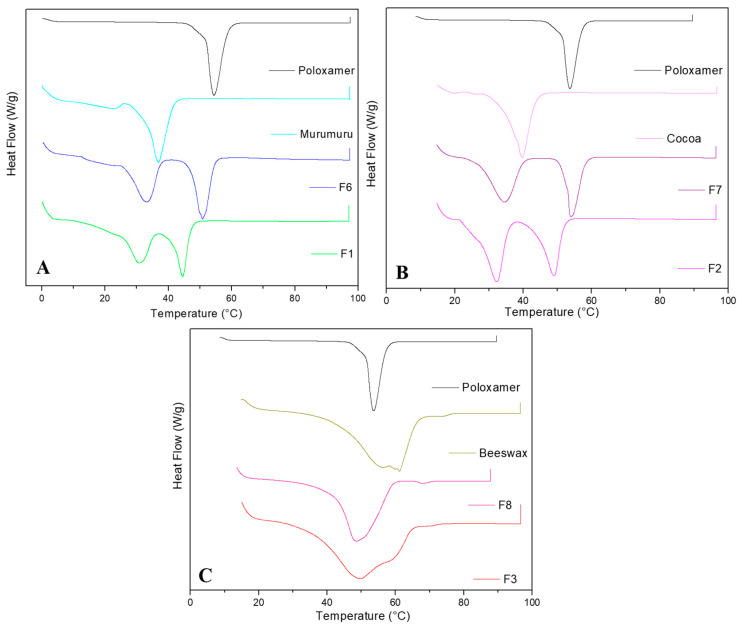
DSC analyses of F1 (**A**), F2 (**B**), F3 (**C**), and their respective controls and excipients.

Figure 4 displays the thermograms of the excipients, NLC controls, and F1 (Figure 4A), F2 (Figure 4B), and F3 (Figure 4C) EO-based NLCs. NLC controls exhibited endothermic peaks between 51 and 54 °C, related to the influence of the melting points of their respective solid lipids (the major component of the formulations) influenced by poloxamer, which exhibited a melting point at 54 °C [9]. The EO-based NLCs composed of murumuru and cocoa butter presented a polymorphic thermal behavior currently observed for the formulations composed of vegetable butter and essential oils [9,22,25] with two mainly endothermic peaks at 31 and 45 °C and 32 and 49 °C, respectively. On the other hand, F3 showed a single peak related to the beeswax melting point at 53 °C [25]. There was no evidence of any degradation or decomposition peaks up to 100 °C in all the analyzed samples.

### 3.5. In Vivo Nanotoxicity Assay on Chicken Embryo Model

**Figure 5:**
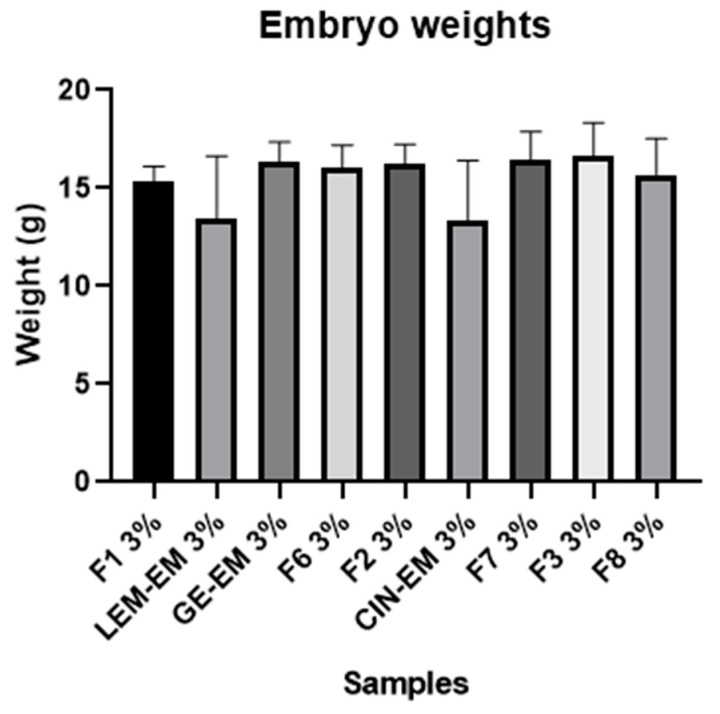
Embryo weight changes after treatment with F1, F6, F7, and F8 formulations and their respective emulsified EO, called LEM-EM, CIN-EM, and GE-EM. None of the formulations presented were statistically significant by ANOVA/Tukey’s test (*p* < 0.05).

A nanotoxicity test was performed using an in vivo chicken embryo model to elucidate the safety of the NLC-based EOs. Emulsified EO, as a positive control, was prepared with both EO and poloxamer at the same concentration as the NLC formulations. The formulation containing 3% geranium EO (F3) and its emulsified form (GE-EM) with 3% EO were the safest systems, as they did not cause mortality (Table 4). In contrast, emulsified cinnamon EO (CIN-EM) and lemongrass EO (LEM-EM) showed mortality rates of 28.57% and 42.85%, respectively, after treatment. The formulations that only contained solid lipids (F6, F7, and F8) did not induce embryonic deaths. None of the formulations resulted in a statistically significant difference (*p* > 0.05) in embryo weight changes (Figure 5). In addition, geranium EO was the safest treatment in both its emulsified (GE-EM) and nanoencapsulated (F3) forms, exhibiting 0% mortality.

## 4. Discussion

Nanoencapsulation is a strategic approach for the physical protection of EO, as it can decrease photodegradation, change physicochemical properties, and increase bioavailability [15]. The NLC formulations were based on the EO with the best in vitro anti-*Campylobacter* activity. Cocoa, murumuru butter, and beeswax were selected as solid lipids of NLC based on their thermal stability, melting points higher than physiological temperature, and ability to successfully encapsulate hydrophobic molecules [27]. The in vitro antimicrobial tests revealed a strictly inverse relationship between the results. The EO with the highest halo inhibition exhibited lower MIC values against *Campylobacter* strains, as observed for F1, F2, and F3. Gram-negative bacteria, such as *Campylobacter*, are more resistant to EO than Gram-positive bacteria, owing to differences in their cell walls. The cell wall structure of Gram-positive bacteria makes it easy for hydrophobic molecules to pass through the cells and act in the cytoplasm and on the cell wall. In contrast, the cell wall of Gram-negative bacteria comprises a 2–3 nm thick peptidoglycan layer, which is thinner than that in the cell walls of Gram-positive bacteria. This peptidoglycan layer is intrinsically linked to the outer membrane (OM) by various lipopolysaccharides and functions as an effective natural barrier. This OM has abundant porins that act as hydrophilic transmembrane channels. Hence, Gram-negative bacteria are essentially resistant to the hydrophobic EOs [28]. EOs are composed of terpenes, polyphenols, terpenoids, and phenylpropenes, among other minor compounds [29]. Lemongrass EO is mainly composed of geranial and neral stereoisomer pairs of citral terpenes, conferring a substantial and less intense lemon aroma to the plant [30]. Citral has antimicrobial properties against various bacteria such as *Staphylococcus aureus, Listeria monocytogenes*, and *Salmonella* Typhimurium [30,31]. Its mechanism of action against bacteria is commonly explained by a decrease in intracellular ATP concentration, which induces hyperpolarization of the microbial cell membrane and reduces bacterial cytoplasmic pH, causing bacterial death. Oregano EO mainly contains carvacrol [32], a monoterpene phenol with antimicrobial activity against various erythromycin-resistant bacteria, such as *Bacillus subtilis*, *Pseudomonas aeruginosa*, and group A *Streptococci* resistant to erythromycin [33,34]. Geranium EO consists of citronellol terpenes, the geranial isomer of citral. These compounds exhibit moderate antimicrobial effects against *S. aureus* and *Escherichia coli* [35]. Cinnamon EO is mainly composed of cinnamaldehyde, a phenylpropene [36] that inhibits *E. coli* growth through cell membrane disruption and oxidative damage [37]. Eugenol is the most abundant component of clove EO [38]. Its mechanism of action involves the presence of a free hydroxyl group that destabilizes the cellular membrane [39]. Fatty acids were the main constituents of the vegetable butters (cocoa and murumuru) used as solid lipids of NLC in this study. The ability of fatty acids to lyse bacterial membranes is attributed to their amphipathic structure, which leads to microbial membrane destabilization, increased cell permeability and lysis, and bacteriostatic and bactericidal activities [40]. Lauric acid, palmitic acid, and oleic acid possess antimicrobial activity against different bacteria, such as *Clostridium perfringens* and *S. aureus* [41]. In contrast, beeswax is composed of fatty acids, esters, diesters, and hydrocarbons. This lipid has shown antimicrobial activity against Gram-positive bacteria, especially *Streptococcus epidermitis* and *Streptococcus pyogenes* [42]. In addition to possessing antimicrobial activities, processing solid and liquid lipids as structural and bioactive matrices of NLC masks organoleptic properties, optimizes their solubility and stability, and decreases photodegradation and volatility, which facilitates their further use in campylobacteriosis treatment. The cinnamon, lemongrass, clove, geranium, and oregano EOs showed excellent antimicrobial activities in this study. Quality control is required for all pharmaceutical formulations. It is determined by evaluating the long-term physicochemical stability based on particle size (nm), PDI, and Zeta potential to elucidate the shelf life of systems [43]. Some biophysical properties of long-term stable nanocolloids, such as particle size < 250 nm (for the administration of invasive routes), PDI values < 0.2, and Zeta potential > ±25 mV are essentials for stable nanocolloids [44]. These parameters were observed for all NLC formulations in the present study, even after 210-day storage at room temperature. The stability of NLC is related to their desired biological activity [45]. In addition, structural characterization was performed by FTIR-ATR, DSC, and FE-SEM analyses. It was observed that the EO encapsulation did not disturb the lipid matrices’ structure, showing more amorphous molecular organization than NLC controls, as expected. The thermal stability was also confirmed, with no evidence of degradation or decomposition of any excipients up to 100 °C. Therefore, the compatibility of the excipients used in the formulations was confirmed, with F1, F2, and F3 being the most promising systems. In the present work, NLC formulations were evaluated using the in vivo nanotoxicity assay on chicken embryos. This alternative model enables the evaluation of drug toxicity at different embryonic incubation times, simulates several administration routes, and is widely used to determine the safety of other antimicrobial nanostructured formulations [46]. Here, F3 (NLC-based geranium EO) was the safest system as it did not show toxicity in any of the analyzed parameters. This formulation was further tested for in vivo efficacy against *C. jejuni* and *C. coli*.

## 5. Conclusions

New therapies that mitigate campylobacteriosis are urgently needed. In this work, a screening of antimicrobial EO was conducted in order to provide bioactive liquid lipids for the preparation of NLC formulations. The formulation composed of geranium EO and beeswax was the most promising anti*-Campylobacter* agent, exhibiting shelf-time for 210 days at 25 °C. Finally, this system did not show any nanotoxicity for all the analyzed parameters, such as the mortality and weight changes in the chicken embryos. Such a formulation was developed at the laboratory scale and is able to be submitted to in vivo efficacy assays in more complex biological models. This work strongly suggests that NLC-based natural lipid developments are versatile alternatives for treating and controlling multidrug-resistant *Campylobacter* strains, also being an effective, safe, and low-cost therapeutic candidate against campylobacteriosis.

## Figures and Tables

**Table 1:** Composition of nanostructured lipid carriers.

NLC	Solid Lipid (%)	Essential Oil (%)	Surfactant (%)
F1	Murumuru butter (8%)	Lemongrass (5%)	Poloxamer (5%)
F2	Cocoa Butter (8%)	Cinnamon (5%)	Poloxamer (5%)
F3	Beeswax (8%)	Geranium (5%)	Poloxamer (5%)
F4	Cocoa Butter (8%)	Clove (5%)	Poloxamer (5%)
F5	Beeswax (8%)	Oregano (5%)	Poloxamer (5%)
F6	Murumuru butter (8%)	-	Poloxamer (5%)
F7	Cocoa Butter (8%)	-	Poloxamer (5%)
F8	Beeswax (8%)	-	Poloxamer (5%)

**Table 2:** Halo inhibition of *Campylobacter* strains treated by essential oils (mm).

Strains Samples EO	131/5 *C. coli*	131/6 *C. coli*	131/7 *C. coli*	30/1 *C. jejuni*	64/5 *C. jejuni*	34,763/3 *C. jejuni*
Cinnamon	95.00 ± 0.00 **	69.00 ± 0.00 **	82.00 ± 0.00 **	86.00 ± 0.00 **	33.00 ± 0.00 **	95.00 ± 2.82 **
Lemongrass	93.50 ± 0.71 **	85.50 ± 0.71 **	80.00 ± 0.00 **	84.00 ± 0.00 **	24.00 ± 0.00 **	106.0 ± 1.41 **
Clove	47.50 ± 0.71 **	53.00 ± 1.41 **	80.50 ± 0.71 **	45.00 ± 0.00 **	15.50 ± 0.71 **	64.00 ± 0.00 **
Geranium	51.00 ± 0.00 **	43.00 ± 0.00 **	64.00 ± 0.00 **	65.50 ± 0.71 **	*	60.00 ± 0.00 **
Oregano	92.00 ± 0.00 **	89.00 ± 0.00 **	95.50 ± 0.71 **	85.00 ± 0.00 **	26.00 ± 1.41 **	83.50 ± 0.71 **
Avocado	*	*	*	*	*	
Tea tree	07.50 ± 0.71 **	*	*	*	*	
Sandalwood	*	*	*	*	10.00 ± 0.00 **	
Citronella	*	*	14.00 ± 0.00 **	08.00 ± 0.00 **	*	
Copaiba	*	12.00 ± 0.00 **	*	07.50 ± 0.71 **	*	
Lavender	15.00 ± 1.41 **	*	*	*	*	
Aloe Vera	*	*	*	*	*	
Garlic	*	*	*	*	08.00 ± 0.00 **	07.50 ± 0.71 **

Note: * there was no inhibition; one-way ANOVA and Tukey’s post hoc tests were used to analyze intragroup statistically significant differences over time; ** *p* < 0.05.

**Table 3:** Determination of the minimum inhibitory concentration (MIC, mg/mL^−1^) of NLC formulations against *Campylobacter* ssp. strains (*n* = 3).

Strains Samples	131/5 C. coli **	131/6 C. coli **	131/7 C. coli **	30/1 C. jejuni **	64/5 C. jejuni **	34,763/3 C. jejuni **
F1	00.23 ± 0.00	00.23 ± 0.00	00.23 ± 0.00	00.23 ± 0.00	24.51 ± 8.49	00.23 ± 0.00
F2	00.23 ± 0.00	00.23 ± 0.00	00.23 ± 0.00	00.23 ± 0.00	01.53 ± 0.53	00.23 ± 0.00
F3	00.20 ± 0.00	00.20 ± 0.00	00.20 ± 0.00	00.23 ± 0.00	39.47 ± 22.79	00.20 ± 0.00
F4	00.20 ± 0.00	00.20 ± 0.00	13.16 ± 0.00	00.47 ± 3.31	*	00.20 ± 0.00
F5	00.26 ± 0.11	00.19 ± 0.00	00.78 ± 0.00	00.19 ± 0.00	04.16 ± 1.81	00.19 ± 0.00
F6	03.33 ± 3.97	00.32 ± 0.14	>62.50 ± 0.00	52.08 ± 18.04	*	10.66 ± 17.8
F7	39.21 ± 16.98	29.41 ± 0.00	39.21 ± 16.98	*	*	29.41 ± 0.00
F8	13.16 ± 0.00	13.16 ± 0.00	>52.63 ± 0.00	*	*	26.32 ± 0.00

Note: F1, lemongrass EO and mumururu butter; F2, cinnamon EO and cocoa butter; F3, geranium EO and beeswax; F4, clove EO and cocoa butter; F5, oregano EO and beeswax; F6, mururumu butter; F7, cocoa butter; and F7, beeswax. All the formulations had poloxamer as a surfactant. * No inhibition. ** *p* < 0.05.

**Table 4:** Mortality rates of chicken embryos after different treatments.

Samples	Mortality (%)
GE-EM	0.00%
F3	0.00%
F6	0.00%
CIN-EM	28.57%
F2	28.57%
F7	14.28%
LEM-EM	42.85%
F1	28.57%
F8	0.00%
NC	25.00%

Note: GE-EM, emulsified geranium EO; F1, lemongrass EO and mumururu butter; CIN-EM, emulsified cinnamon EO; F2, cinnamon EO and cocoa butter; LEM-EM, emulsified lemongrass EO; F3, geranium EO and beeswax; NC, saline solution as negative control; F6, mururumu butter; F7, cocoa butter; and F8, beeswax. All formulations employed poloxamer as a surfactant. None of the formulations showed statistically significant differences, as determined using the chi-square test (*p* > 0.05).

## Data Availability

The data used in this study are available from the corresponding author upon request.
